# Supplementary material for: CXCR3 Expression and Genome-Wide 3′ Splice Site Selection in the TCGA Breast Cancer Cohort
Source: Life (Basel). 2021 Jul 26;11(8):746. doi: 10.3390/life11080746 (PMC8398780; doi:10.3390/life11080746)
Supplement: Supplementary file 1 [file life-11-00746-s001.zip › life-1288481-supplementary.pdf]

life

Supplementary Material of CXCR3 Expression and Genome-Wide 3′ Splice Site Selection in the TCGA Breast Cancer Cohort

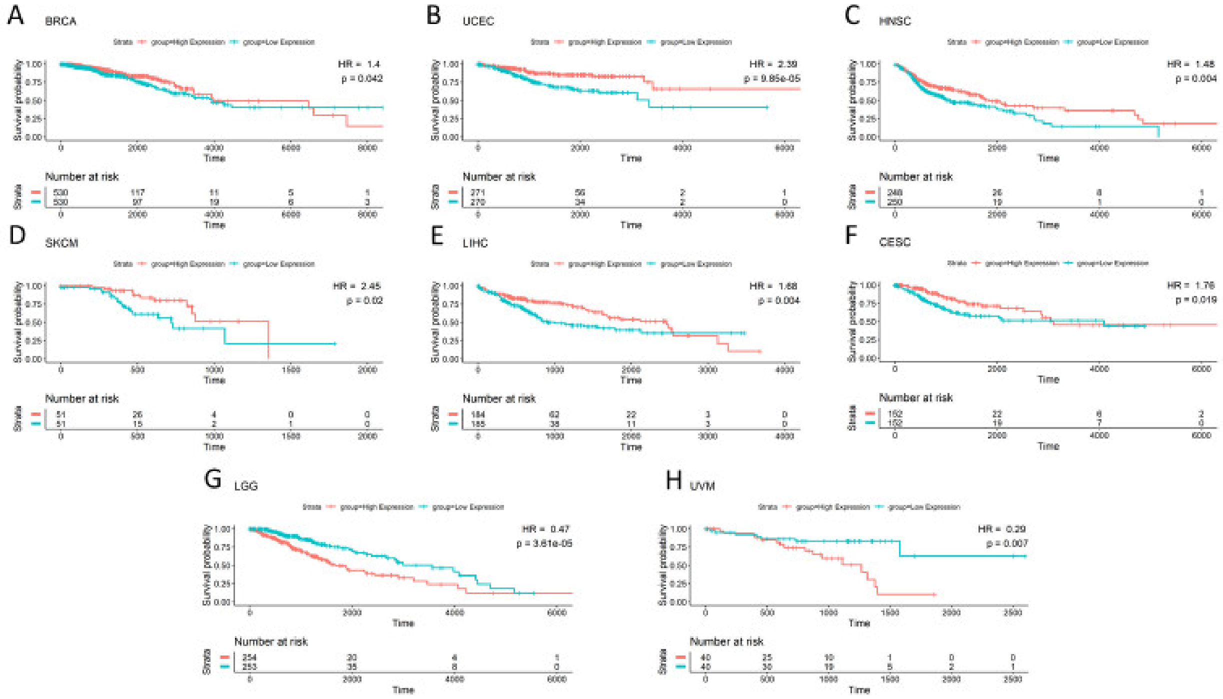

Figure S1. Significant survival correlations with CXCR3 gene expression. Kaplan–Meier survival curves of patients split at median CXCR3 expression into 2 groups, low(blue) and high (red). Top two rows (**A**–**F**) show cohorts where high CXCR3 expression was correlated with better survival (Cox proportional analysis, *p*-value <0.05, HR >1). Bottom row (**G**,**H**) shows cohorts where low CXCR3 expression was correlated with better survival (Cox proportional analysis, *p*-value < 0.05, HR < 1).

Table S1. Pan-Cancer CXCR3 Splicing Results.

Cohort	Cox Survival P-value	Unpaired Tumor vs. Normal P-value
BRCA	0.64	Not Enough Samples
OV	0.54	Not Enough Samples
LUAD	0.81	Not Enough Samples
KIRC	0.79	Not Enough Samples
HNSC	0.83	Not Enough Samples
THCA	0.085	1
LUSC	0.09	0.948768785
SKCM	0.84	Not Enough Samples
COAD	0.99	0.809655052
STAD	0.43	Not Enough Samples
BLCA	0.63	Not Enough Samples
CESC	0.62	Not Enough Samples
SARC	0.32	Not Enough Samples
PAAD	0.95	Not Enough Samples

READ	0.4	Not Enough Samples
TGCT	0.19	Not Enough Samples
THYM	0.67	Not Enough Samples
MESO	0.14	Not Enough Samples
DLBC	0.42	Not Enough Samples

Not Enough Samples for Survival: <5 tumor samples following filtering (sum of ≥20 reads across both junctions). Not Enough Samples for Tumor vs. Normal: <5 tumor sample and <5 normal samples following filtering (sum of ≥20 reads across both junctions). Cancers that are absent in table did not have enough samples for either analysis.

Table S2. Pan-Cancer CXCR3 Gene Expression Results.

Cohort	Cox Survival P-Value	Tumor vs. Normal P-value
BRCA	0.04	<0.0001
GBM	0.53	Not Enough Samples
OV	0.25	NA
LUAD	0.5	0.395
UCEC	< 0.0001	0.482
KIRC	0.076	0.000
HNSC	0.004	0.507
LGG	< 0.0001	Not Enough Samples
THCA	0.75	0.693
LUSC	0.76	0.0001
PRAD	0.82	0.938
SKCM	0.017	Not Enough Samples
COAD	0.88	0.898
STAD	0.5	0.006
BLCA	0.45	0.258
LIHC	0.0034	0.862
CESC	0.018	Not Enough Samples
KIRP	0.92	0.034
SARC	0.0036	Not Enough Samples
ESCA	0.67	0.078
PAAD	0.96	Not Enough Samples
PCPG	0.4	NA
READ	0.2	0.250
TGCT	0.78	Not Enough Samples
THYM	0.058	Not Enough Samples
KICH	0.82	0.092
ACC	0.41	Not Enough Samples
MESO	0.18	Not Enough Samples
UVM	0.004	Not Enough Samples
DLBC	0.54	Not Enough Samples
UCS	0.49	Not Enough Samples
CHOL	0.31	0.820
